# Integrating Livestock Grazing and Sympatric Takin to Evaluate the Habitat Suitability of Giant Panda in the Wanglang Nature Reserve

**DOI:** 10.3390/ani11082469

**Published:** 2021-08-23

**Authors:** Xiaoyu Chen, Xiaorong Wang, Junqing Li, Dongwei Kang

**Affiliations:** 1 School of Ecology and Nature Conservation, Beijing Forestry University, Beijing 100083, China; xiaoyuchen@bjfu.edu.cn (X.C.); lijq@bjfu.edu.cn (J.L.); 2 Wanglang National Nature Reserve Administration Bureau, Pingwu County, Mianyang 622553, China; xiaorongwang2021@163.com

**Keywords:** suitable habitat, habitat overlap, habitat area, habitat selection, species protection

## Abstract

**Simple Summary:**

Giant pandas are facing the threat of habitat degradation. Both grazing livestock and sympatric animals have certain impacts on the giant panda habitat. This study evaluated the habitat suitability of giant panda in Wanglang Nature Reserve by simultaneously investigating livestock grazing and sympatric takin. Results for the giant panda habitat in Wanglang are not optimistic, and the extensive overlap of suitable habitats for livestock, takin and panda makes the situation worse.

**Abstract:**

Habitat suitability provides essential information for the management of protected species. However, studies that jointly consider the impacts of human disturbance and sympatric animals in habitat suitability assessments of giant panda are limited, which may overestimate the habitat status. To address this issue, we evaluated the habitat suitability of giant panda in Wanglang Nature Reserve by simultaneously investigating livestock grazing and sympatric takin via MAXENT, a new attempt at the assessment of the habitat suitability of giant panda. We focused on describing the habitat suitability of giant panda and determining the habitat overlap between livestock, takin, and panda to evaluate the impacts of livestock grazing and sympatric takin on the suitable giant panda habitat. Results revealed that only 16.33% of the area in Wanglang was suitable giant panda habitat, of which 67.66% was shared by livestock, and 97.99% of the remaining suitable panda habitat not shared by livestock was revealed to be shared by takin. The results indicate an unfavorable habitat status of giant panda in Wanglang, with the potential extensive habitat overlap between livestock, takin and panda exerting further pressure. Thus, to effectively protect giant pandas and their habitats, grazing activity should be controlled. Furthermore, to accurately protect sympatric animals, the monitoring of panda and takin activities in the overlapping areas must be maintained.

## 1. Introduction

Habitat degradation is a threat to the survival of wild animals [1]. As one of the national treasures of China, the survival of giant panda (*Ailuropoda melanoleuca*) has been largely impacted by habitat degradation due to anthropogenic activities such as livestock grazing, roads, logging, and herb collection, as well as natural factors such as earthquakes and landslides [2] (p. 130). To restore the degraded giant panda habitats, the causes of degradation must be identified and a habitat assessment should be comprehensively implemented.

Livestock grazing is a major and common human disturbance of giant panda habitats [2] (p. 130) via occupation, disturbance, and defecation, and, as a consequence, these habitats are avoided by giant pandas [3]. Previous studies on the effects of grazing generally focus on habitat usage and preference, space-use patterns and activity rhythms [4,5,6], while few studies consider the suitability and fragmentation of habitats [7]. Thus, our understanding of the large-scale impacts of grazing are limited, which consequently restricts the effective control and management of this disturbance.

Many wild animals are sympatric with giant panda, such as takin (*Budorcas tibetanus*), red panda (*Ailurus fulgens*), and golden monkey (*Rhinopithecus roxellanae*), with several species competing with giant pandas in terms of food and territory [8] (pp. 30–39), [9]. Previous research on giant panda and its sympatric animals generally focused on exploring the reasons behind their coexistence [5,6,10], while studies on the impacts of the existence of sympatric animals on giant pandas and their habitats is so limited that it would restrict the protection of multiple sympatric species, which is the key for biodiversity conservation.

Among sympatric animals of giant panda, takin is the main competitor of the giant panda in terms of food resources [11] and has many similarities with the giant panda in terms of habitat utilization [12,13], it therefore poses a potential threat to the habitat selection of giant panda. Therefore, the impact of takin on the giant panda habitat should not be ignored.

Habitat suitability assessments provide essential information for habitat protection and restoration [14]. Previous habitat suitability assessments of giant panda were based on natural factors (e.g., elevation, slope, aspect, forest cover type, and bamboo) [15,16,17] and also considered human disturbances (e.g., livestock grazing, roads, timber harvest, crop, herb collection and residential areas) [14,18,19,20]. However, few studies investigate the impacts of sympatric animals [21], with even less jointly considering human disturbance and sympatric animals. Ignoring these two factors may overestimate the habitat status of giant panda.

To accurately grasp the habitat status of giant panda, in the current study we evaluated the habitat suitability of giant panda in Wanglang Nature Reserve based on natural conditions by jointly considering livestock grazing and sympatric takin. This is a new attempt in the assessment of habitat suitability of giant panda. More specifically, the objectives of this study are to: (1) describe the habitat suitability characteristics of giant panda under natural conditions; (2) evaluate the possible impact of livestock grazing on the habitat suitability of giant panda; and (3) predict the habitat overlap between sympatric takin and giant panda. Considering that (a) livestock grazing is widespread in giant panda habitat [2] (p. 130); (b) livestock and giant panda exhibit many similarities in the habitat selection process [22]; and (c) takin are important companions of giant panda [11,23,24], we hypothesize that livestock and takin have reduced the suitable giant panda habitat in size through habitat overlap.

## 2. Materials and Methods

### 2.1. Study Area

Wanglang Nature Reserve (103°57′–104°11′ E, 32°49′–33°03′ N) is one of the first four giant panda nature reserves established in China [8] (p. 82). The total area of Wanglang is approximately 322 km^2^, 41.58% of which is forest. Wanglang is a key area for giant panda in the Minshan Mountains [25] and is home to 28 giant pandas [2] (p. 10). A large number of livestock (including cattle and horse) from the surrounding communities are free-ranging and scattered in the reserve [7]. Moreover, 25 protected wild animals live in Wanglang, such as takin, golden monkey, and black bear (*Ursus thibetanus*) [23].

### 2.2. Data Sources

**Figure 1:**
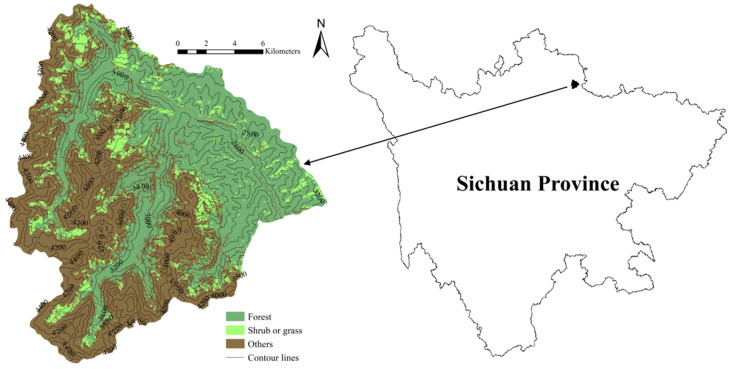
The location and forest cover type distribution map of Wanglang Nature Reserve.

**Figure 2:**
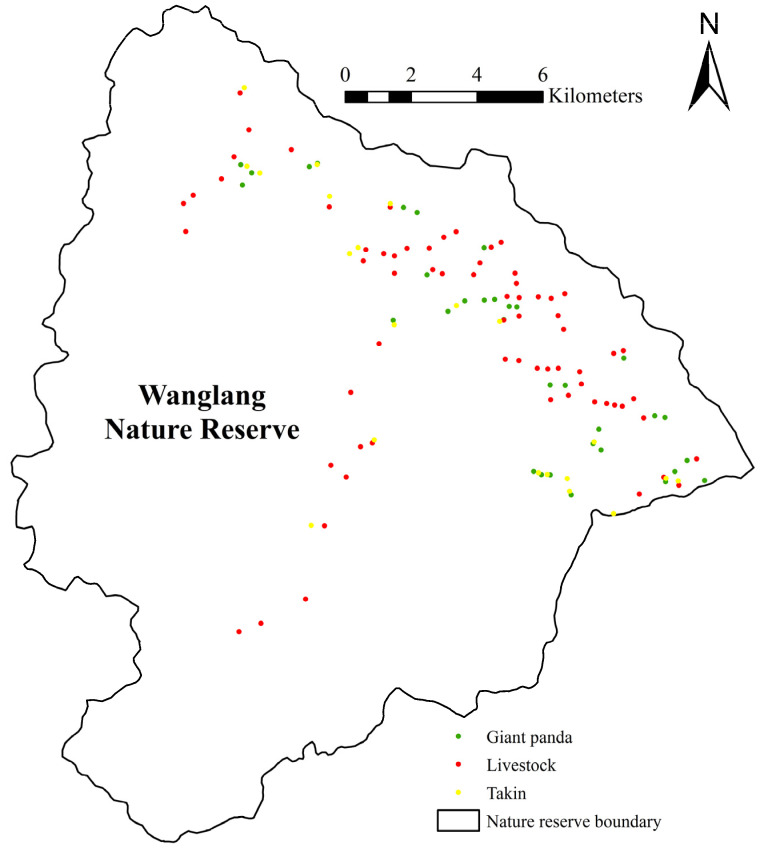
Distribution of the giant panda, livestock, and takin presence data.

We employed elevation, slope, and forest-cover type to evaluate the habitat suitability of Wanglang. These environmental factors are important indicators that can reflect habitat quality and suitability, and were widely adopted in habitat assessments [4,15,26]. Elevation and slope data were extracted from digital elevation model (DEM) data with a grid size of 30 m × 30 m (DEM) using ArcGIS 10.2. For forest-cover type, we interpreted and classified Landsat 8 OLI_TIRS imagery (collected on July 16, 2013) and divided the forest-cover type into three categories, namely forest, shrub or grass, and others (such as bare land, water bodies, etc.) with a classification accuracy of 87.5% (Figure 1). We obtained the DEM data (accessed on 1 June 2020) and Landsat imagery (accessed on 11 June 2019) from the geospatial data cloud site of the Computer Network Information Center, Chinese Academy of Sciences (http://www.gscloud.cn, accessed on 20 August 2021). The presence of giant panda, livestock (cattle and horse), and takin was determined from field surveys conducted during April–May 2012 and March–May 2013 [27,28]. Specifically, 40 survey transects with a length of not less than 2 km were set up to record the location of the feces of giant panda, livestock, or takin encountered in the transects [28], resulting in a total of 75 giant panda, 130 livestock, and 45 takin presence data. In order to avoid the effects of spatial autocorrelation on the model prediction, we employed the ArcGIS SDMtoolbox to eliminate redundant presence data with a distance of less than 300 m for each category [7,29]. A total of 33 giant panda, 67 livestock, and 21 takin presence data were retained for the subsequent analysis (Figure 2, Tables S1–S3).

### 2.3. Habitat Suitability Assessment of Giant Panda

To describe the habitat suitability of giant panda, MAXENT 3.4.1 and ArcGIS 10.2 were employed to predict the distribution and area of giant panda habitats across different suitability grades [30]. First, we converted the three environmental variables of elevation, slope, and forest-cover type into ASCII format. Following this, 75% of the giant panda presence data was selected for model building, while the remaining 25% were taken to test the model. MAXENT was run repeatedly 10 times in order to select the model with the highest test AUC (Area under the Receiving Operating Characteristic Curve (ROC)) value [31]. We then classified the prediction results using Jenks natural break optimization and divided them into three categories: suitable habitat, generally suitable habitat, and unsuitable habitat [32]. Finally, we divided the suitable habitat area by 28 to obtain the average suitable habitat area per giant panda.

### 2.4. Habitat Overlap between Livestock and Giant Panda

The potential impacts of livestock grazing on giant panda habitat suitability were evaluated by analyzing the habitat overlap of livestock and giant panda. First, we predicted the distribution and area of livestock habitats across varying suitability grades following the method adopted for the giant panda habitat assessment. We then overlaid the suitable habitat maps of livestock and panda to determine the habitat overlap map and calculated the area and proportion of the suitable panda habitat shared by livestock. To clarify the impact of livestock grazing, we focused on the suitable panda habitats that were also suitable habitats for livestock.

### 2.5. Habitat Overlap between Takin and Giant Panda

To evaluate the possible impact of sympatric takin on the habitat suitability of giant panda under the condition of livestock grazing, we further analyzed the habitat overlap of takin and panda. First, we predicted the distribution and area of takin habitats across varying suitability grades following the method adopted for the giant panda habitat assessment. We then overlaid the suitable habitat maps of takin and panda to obtain the corresponding habitat overlap map and calculated the area and proportion of the suitable panda habitat shared by takin. To clarify the impact of the presence of takin, we focused on the suitable panda habitats not shared by livestock that were also suitable habitats of takin.

## 3. Results

### 3.1. Habitat Suitability Characteristics of Giant Panda

**Figure 3:**
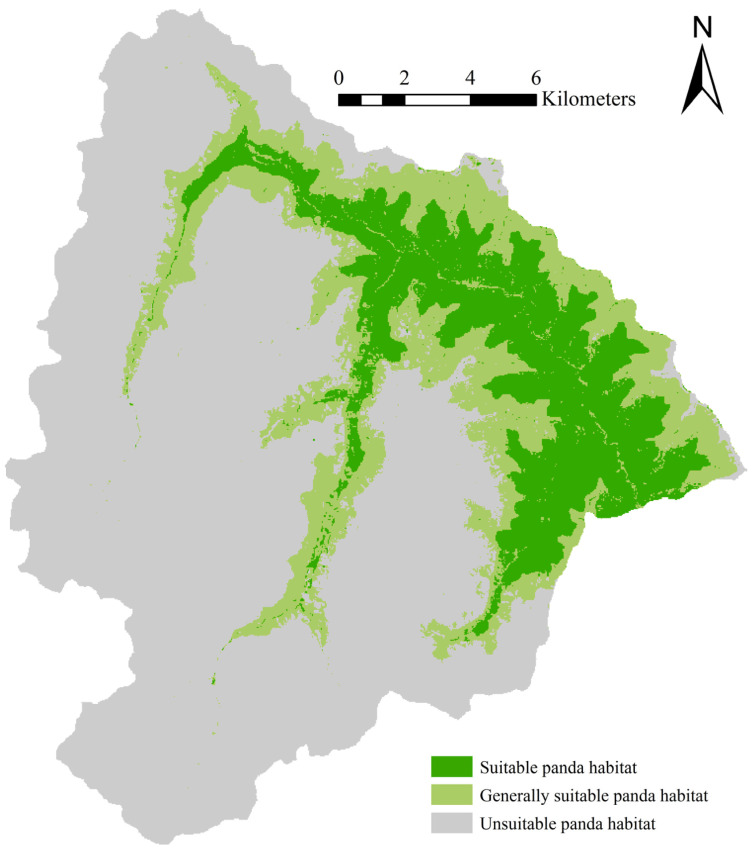
Giant panda habitat across varying suitable grades in Wanglang Nature Reserve.

The ROC verification results for giant panda reveal training and testing AUC values of 0.903 and 0.905, respectively, indicating a high model prediction accuracy. The MAXENT results for giant panda determine the areas of suitable habitat, generally suitable habitat, and unsuitable habitat as 52.583 km^2^, 59.394 km^2^, and 210.010 km^2^, accounting for 16.33%, 18.45%, and 65.22% of the total area of Wanglang, respectively (Figure 3). The average suitable habitat area per panda was 1.878 km^2^.

### 3.2. Suitable Giant Panda Habitat Shared by Livestock

**Figure 4:**
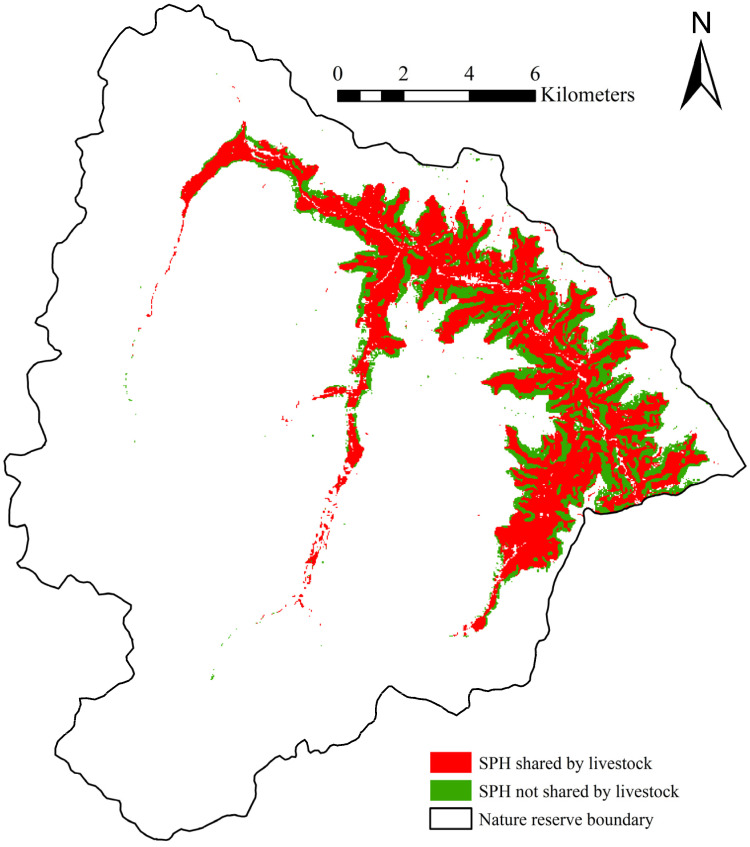
Suitable giant panda habitat shared by livestock (SPH: suitable giant panda habitat).

The ROC verification results for livestock determined training and testing AUC values of 0.917 and 0.942, respectively, indicating a high model prediction accuracy. A wide range of habitat overlap was observed between livestock and giant panda. More specifically, 67.66% (35.580 km^2^ of 52.583 km^2^) of the suitable panda habitat was shared by livestock, and 32.34% (17.004 km^2^ of 52.583 km^2^) of the suitable panda habitat was not shared by livestock (Figure 4).

### 3.3. Suitable Giant Panda Habitat Shared by Takin

**Figure 5:**
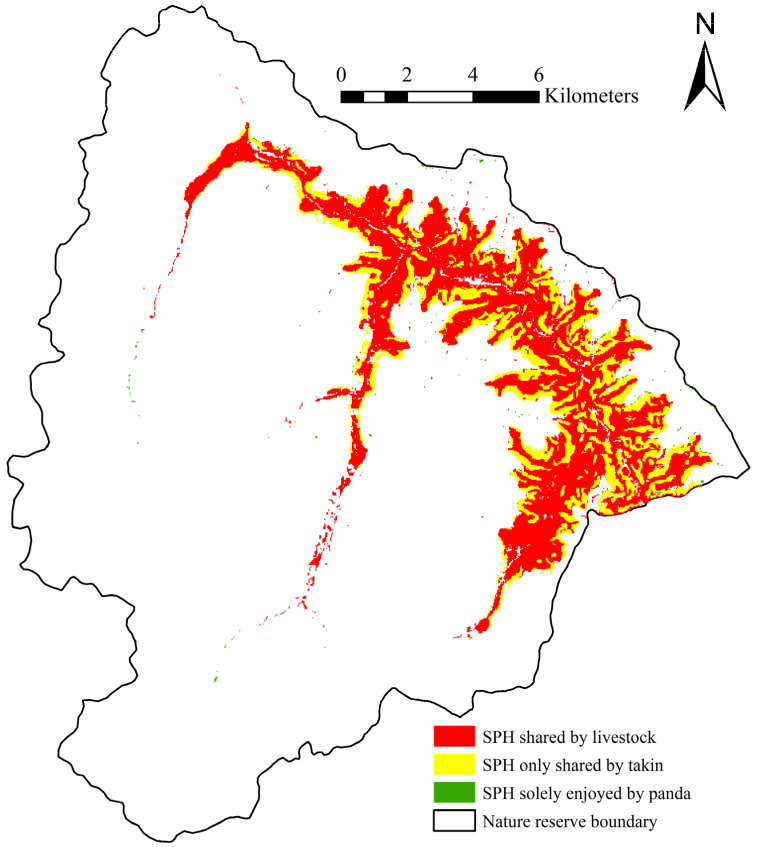
Suitable giant panda habitat shared by livestock and takin (SPH: suitable giant panda habitat).

The ROC verification results for takin reveal training and testing AUC values of 0.869 and 0.885, respectively, indicating a high model prediction accuracy. 97.99% (16.663 km^2^ of 17.004 km^2^) of suitable panda habitats not shared by livestock were shared by takin, and only 2.01% (0.341 km^2^ of 17.004 km^2^) was solely enjoyed by panda. More specifically, only 0.65% (0.341 km^2^ of 52.583 km^2^) of the total suitable panda habitat was solely enjoyed by panda (Figure 5). Furthermore, 96.77% (34.431 km^2^ of 35.580 km^2^) of the total suitable panda habitat shared by livestock was also suitable habitat for takin.

## 4. Discussion

### 4.1. Habitat Status of Giant Pandas under Natural Conditions

Suitable giant panda habitats denote habitats that are more likely to provide high-quality survival conditions for giant panda [18]. Our model, established by elevation, slope and forest-cover type, determined the proportion of suitable giant panda habitat in Wanglang to be 16.33%, and thus the average suitable habitat area per panda was only 1.878 km^2^. This is less than 3.26 km^2^, the lower limit home range of giant panda determined in previous studies [33,34] and thus indicates an unfavorable habitat status for giant panda in Wanglang.

Habitat suitability assessment results can be affected by multiple factors, such as assessment methods, assessment indicators, etc. [35,36]. The suitable habitat area is likely to decrease as the number of assessment indicators increase due to the greater amount of constraints [14]. In the current study, we just consider elevation, slope, and forest-cover type; additional indicators, such as bamboo, may have an important effect on the habitat suitability and assessment results [37,38]. Thus, the actual suitable habitat area may be lower than that estimated by the proposed model.

### 4.2. Livestock Grazing Threatens Habitat Quality and Habitat Selection of Giant Panda

Livestock are widely distributed within giant panda habitats and frequently occur across many nature reserves [2] (p. 130), [39]. Moreover, giant pandas avoid habitats disturbed by grazing [40]. Livestock disturb giant panda food resources by eating and trampling across bamboo [7,41]. Furthermore, livestock also affect the soil structure and the growth of understory plants [5]. In this study, we determined a wide range of overlap between the suitable habitats of panda and livestock. As a consequence, the habitat quality of giant panda may be reduced following its disturbance by livestock, thus weakening the habitat status of giant panda.

The habitat usage and daily activity trends of livestock and giant panda typically overlap [4,22,42,43]. Furthermore, livestock travel longer distances each day compared to giant panda [5], with a greater activity intensity and active time [6,19]. Based on the possible habitat overlap between panda and livestock and the avoidance of grazing by giant panda [42], we suggest that livestock grazing poses a potential threat to the habitat selection of giant panda.

Livestock grazing is a common human disturbance and grazing activities require urgent control. Furthermore, the disturbed habitats should be restored.

### 4.3. Potential Effects of Takin on the Habitat Selection of Giant Pandas

As an important companion of giant panda [44,45], takin largely occur sympatricly in giant panda habitats [12,13,24,46]. In the current study, almost all of the suitable panda habitats not shared by livestock were shared by takin. This indicates that these habitats were also available for takin, resulting in competition between giant panda and takin for these habitats. Furthermore, the majority of suitable panda habitats shared by livestock were also identified as suitable habitat for takin. This can further compress the available habitat range of giant panda.

In Tangjiahe Nature Reserve, giant pandas avoid habitats occupied by the takin populations with a large number of individuals [8] (pp. 30–39). In Wanglang, although the specific number of takin is unclear, related studies have found that large-sized species such as takin significantly affected the occurrence of giant pandas [47]. Takin may compete with giant pandas for food [48], and also exhibit similar habitat use patterns to giant panda [10,12,13,46]. Thus, we propose the potential effect of takin on the habitat selection of giant pandas via bamboo consumption and habitat occupation.

Unlike cattle and horse, takin is a protected animal. Considering the potential habitat overlap between takin and panda, the monitoring of these two species should be strengthened in the overlapping areas in order to provide a scientific basis for the adoption of effective targeted protection strategies.

### 4.4. Limitations of the Study

This study evaluated the habitat suitability of giant panda in Wanglang Nature Reserve using elevation, slope, and forest-cover type as variables. Based on the sample size obtained from the field survey, this issue has been clearly explained. However, considering that a large sample size may be more convincing, to further optimize the model, it should be considered to increase the sample size, especially the takin presence data. The lack of bamboo distribution data may make the suitable habitat area of giant panda overestimated, but nonetheless the results indicate an unfavorable habitat status of giant panda in Wanglang. We mainly studied the impact of grazing livestock on the habitat suitability of giant pandas through habitat overlap on a large scale. However, additional human disturbances were not considered in this study such as hunting, herb collection, and tourism [25,49,50], all of which damage habitats and may reduce suitable giant panda habitat areas. As for the impact of takin on the giant panda habitat, this study only analyzed the overlap of suitable habitats for takin and giant panda, and it may be worthwhile to conduct more in-depth research in the future.

## 5. Conclusions

In the current study, we evaluated the habitat suitability of giant panda in Wanglang under natural conditions by jointly considering livestock grazing and the presence of sympatric takin. Under natural conditions, both the area and proportion of suitable panda habitat were determined to be limited. Following the introduction of livestock grazing and sympatric takin, the majority of the suitable panda habitat was observed to be shared by livestock and takin, with just a small proportion (0.65%) of suitable panda habitat solely enjoyed by giant panda. In order to effectively protect giant panda and their habitats, it is urgent to make preparations in advance to prevent livestock from entering the relevant areas. Specifically, these preparations should aim to control the number and range of livestock, and even drive them out of the reserve. Furthermore, the effective protection of sympatric animals requires the stronger monitoring of panda and takin activities in their overlapping areas.

## Data Availability

The data presented in this study are available in the Supplementary Materials.

## Supplementary Materials

The following are available online at https://www.mdpi.com/article/10.3390/ani11082469/s1, Table S1: Location of giant panda presence data, Table S2: Location of livestock presence data, Table S3: Location of takin presence data.

## References

[1] Wei F., Nie Y., Miao H., Lu H., Hu H. (2014). Advancements of the researches on biodiversity loss mechanisms. Chin. Sci. Bull..

[2] Sichuan Forestry Department (2015). The Pandas of Sichuan: The 4th Survey Report on Giant Panda in Sichuan Province.

[3] Kang D. (2021). A review of the impacts of four identified major human disturbances on the habitat and habitat use of wild giant pandas from 2015 to 2020. Sci. Total Environ..

[4] Kang D., Zhao L., Song G. (2011). Competition relationship between giant panda and livestock in Wanglang National Nature Reserve, Sichuan. J. Northeast For. Univ..

[5] Zhou S., Hull V., Zhang J., Huang J., Liu D., Huang Y., Li D., Zhang H. (2016). Comparative space use patterns of wild giant pandas and livestock. Acta Theriol. Sin..

[6] Zhou S., Zhang J., Hull V., Huang J., Liu D., Zhou J., Sun M., Zhang H. (2019). Comparative activity patterns of wild giant pandas and livestock. Acta Ecol. Sin..

[7] Li B.V., Pimm S.L., Li S., Zhao L., Luo C. (2017). Free-ranging livestock threaten the long-term survival of giant pandas. Biol. Conserv..

[8] Zhao X. (2006). Giant Pandas: Natural Heritage of the Humanity.

[9] Hu J., Wu P. (2007). Large-scale and Middle-scale Coexistent Mammals of Giant Panda in Xiaoxiangling Mountains. Sichuan J. Zool..

[10] Kang D., Yang H., Li J., Chen Y. (2013). Can conservation of single surrogate species protect co-occurring species?. Environ. Sci. Pollut. Res..

[11] National Forestry and Grassland Administration (2021). The 4th National Survey Report on Giant Panda in China.

[12] Wei W., Huang Y., Zhou H., Yuan S., Zhou Z., Nie Y., Zhang Z. (2017). Microhabitat separation between giant panda and golden takin in the Qinling Mountains and implications for conservation. North-West. J. Zool..

[13] Li S., Kang D., Li J., Huang J., Song Z. (2017). Comparison of habitat use of giant panda, takin and Sichuan golden monkey. J. Northeast For. Univ..

[14] Wu P., Liu X., Shao X., Zhu Y., Cai Q. (2013). GIS application in evaluating the potential habitat of giant pandas in Guanyinshan nature reserve, Shaanxi Province. J. Environ. Inform..

[15] Liu J., Linderman M., Ouyang Z., An L., Yang J., Zhang H. (2001). Ecological degradation in protected areas the case of Wolong nature reserve for giant pandas. Science.

[16] Liu X., Skidmore A.K., Bronsveld M.C. (2006). Assessment of giant panda habitat based on integration of expert system and neural network. Chin. J. Appl. Ecol..

[17] Xu W., Ouyang Z., Jiang Y., Zheng H., Liu J. (2006). Assessment of giant panda habitat in the Daxiangling Mountain Range, Sichuan, China. Biodivers. Sci..

[18] Ouyang Z., Liu J., Xiao H., Tan Y., Zhang H. (2001). An assessment of giant panda habitat in Wolong Nature Reserve. Acta Ecol. Sin..

[19] Zhang J., Hull V., Ouyang Z., Li R., Connor T., Yang H., Zhang Z., Silet B., Zhang H., Liu J. (2017). Divergent responses of sympatric species to livestock encroachment at fine spatiotemporal scales. Biol. Conserv..

[20] He K., Dai Q., Gu X., Zhang Z., Zhou J., Qi D., Gu X., Yang X., Zhang W., Yang B. (2019). Effects of roads on giant panda distribution: A mountain range scale evaluation. Sci. Rep..

[21] He M., Chen L., Luo G., Gu X., Wang G., Ran J. (2018). Suitable habitat prediction and overlap analysis of two sympatric species, giant panda (*Ailuropoda melanoleuca*) and Asiatic black bear (*Ursus thibetanus*) in Liangshan Mountains. Biodivers. Sci..

[22] Ran J., Liu S., Wang H., Sun Z., Zeng Z., Liu S. (2003). Habitat selection by giant pandas and grazing livestock in the Xiaoxiangling Mountains of Sichuan Province. Acta Ecol. Sin..

[23] Tian C., Li J., Yang X., Yu L., Yuan D., Li Y. (2018). Preliminary surveys of wild animals using infrared camera in Wanglang National Nature Reserve, Sichuan Province. Biodivers. Sci..

[24] Gao H., Guan T., Zhu D., Li W., Zhou F., Zhao D., Li C., Zhang S.L. (2020). Assessment of effective conservation of the Sichuan takin by giant panda reserves through functional zoning. Integr. Zool..

[25] Chen Y., Jiang S., Zhao L., Huang J. (2003). The trend of human interference activities and management countermeasures in Wanglang Nature Reserve, Sichuan. Sichuan J. Zool..

[26] Gong X., Fu Q., Wang L., Yang B., Zhang Q., Zhang Y. (2020). Habitat suitability assessment and overlap analysis of *Rusa unicolor* and *Budorcas taxicolor* in Anzihe Reserve, Sichuan Province. Acta Ecol. Sin..

[27] Duan L., Kang D., Wang X., Yang H., Li J. (2014). Comparative habitat use by giant pandas in primary and secondary forests in Wanglang Nature Reserve. J. Biol..

[28] Kang D. (2015). Research on the Habitat Selection of Giant Pandas. Ph.D. Thesis.

[29] Brown J. (2014). SDMtoolbox: A python-based GIS toolkit for landscape genetic, biogeographic and species distribution model analyses. Methods Ecol. Evol..

[30] Phillips S., Anderson R., Schapire R. (2006). Maximum entropy modeling of species geographic distributions. Ecol. Model..

[31] Wang R., Yang H., Luo W., Wang M., Lu X., Huang T., Zhao J., Li Q. (2019). Predicting the potential distribution of the Asian citrus psyllid, Diaphorina citri (*Kuwayama*), in China using the MaxEnt model. PeerJ.

[32] Luo C., Xu W., Zhou Z., Ouyang Z., Zhang L. (2011). Habitat prediction for forest musk deer (*Moschus berezovskii*) in Qinling mountain range based on niche model. Acta Ecol. Sin..

[33] Hu J., Schaller G.B. (1985). Wolong’s Giant Panda.

[34] Pan W., Lv Z., Zhu X., Wang D., Wang H., Long Y., Fu D., Zhou X. (2001). Chance for Lasting Survival.

[35] Wang X., Xu W., Ouyang Z., Liu J., Xiao Y., Chen Y., Zhao L., Huang J. (2008). The application of Ecological-Niche factor analysis in giant pandas (*Ailuropoda melanoleuca*) habitat assessment. Acta Ecol. Sin..

[36] Yang M., Ouyang Z., Xu W., Long Q., Xie Q. (2017). Assessment of the Potential Suitable Habitat and the Actual Use Habitat of Giant Pandas in Wolong. J. Sichuan Agric. Univ..

[37] Liu X., Toxopeus A.G., Skidmore A.K., Shao X., Dang G., Wang T., Prins H.H.T. (2005). Giant panda habitat selection in Foping Nature Reserve, China. J. Wildlife Manag..

[38] Hull V., Roloff G., Zhang J., Liu W., Zhou S., Huang J., Xu W., Ouyang Z., Zhang H., Liu J. (2014). A synthesis of giant panda habitat selection. Ursus.

[39] State Forestry Administration (2006). The 3rd National Survey Report on Giant Panda in China.

[40] Wei W., Swaisgood R.R., Dai Q., Yang Z., Yuan S., Owen M.A., Pilfold N.W., Yang X., Gu X., Hong Z. (2018). Giant panda distributional and habitat-use shifts in a changing landscape. Conserv. Lett..

[41] Zhou S., Zhang J., Hull V., Huang J., Liu D., Xie H., Zou X., Zhang H. (2021). Comparison of the bamboo-grazing behavior of giant pandas and livestock. Chin. J. Appl. Environ. Biol..

[42] Hull V., Zhang J., Zhou S., Huang J., Viña A., Liu W., Tuanmu M., Li R., Liu D., Xu W. (2014). Impact of livestock on giant pandas and their habitat. J. Nat. Conserv..

[43] Tian C., Zhang Y., Liu Z., Dayananda B., Fu X., Yuan D., Tu Z., Luo C., Li J. (2020). Temporal niche patterns of large mammals in Wanglang National Nature Reserve, China. Glob. Ecol. Conserv..

[44] Zeng Z., Zhong W., Song Y., Li J., Zhao L., Gong H. (2003). Present status of studies on eco-biology of takin. Acta Theriol. Sin..

[45] Gong T., Ge B., Chen L., You Z., Tang Z., Liu H., Song Y. (2015). Home range and fidelity of Sichuan takin. Acta Ecol. Sin..

[46] Wei W., Han H., Zhou H., Cao S., Zhang Z. (2018). Microhabitat use and separation between giant panda (*Ailuropoda melanoleuca*), takin (*Budorcas taxicolor*), and goral (*Naemorhedus griseus*) in Tangjiahe Nature Reserve, China. Folia Zool..

[47] Liu Z., Dayananda B., Jeffree R.A., Tian C., Zhang Y., Yu B., Zheng Y., Jing Y., Si P., Li J.Q. (2020). Giant panda distribution and habitat preference: The influence of sympatric large mammals. Glob. Ecol. Conserv..

[48] Hu J. (2001). Research on the Giant Pandas.

[49] Zhang T., Deng D., Yan W. (2011). Effect of giant panda ecotourism on giant pandas and their habitats and strategies. J. Sichuan Forestry Sci. Technol..

[50] Zeng Y., Zhang J., Hull V. (2019). Mixed-method study on medicinal herb collection in relation to wildlife conservation: The case of giant pandas in China. Integr. Zool..

