# Supplementary material for: Integrating Livestock Grazing and Sympatric Takin to Evaluate the Habitat Suitability of Giant Panda in the Wanglang Nature Reserve
Source: Animals (Basel). 2021 Aug 23;11(8):2469. doi: 10.3390/ani11082469 (PMC8388666; doi:10.3390/ani11082469)
Supplement: Supplementary file 1 [file animals-11-02469-s001.zip › animals-1288713-supplementary.pdf]

Supplementary Material

Table S1 Location of giant panda presence data.

Table S2 Location of livestock presence data.

Table S3 Location of takin presence data.

Table S1 Location of giant panda presence data.

ID	Longitude(°)	Latitude(°)	ID	Longitude(°)	Latitude(°)
1	104.09093	32.96144	18	104.02318	33.00099
2	104.10270	32.96457	19	104.02380	32.99542
3	104.09634	32.96433	20	104.02675	32.99877
4	104.11330	32.96280	21	104.04813	33.00152
5	104.07317	32.95887	22	104.04548	33.00061
6	104.12173	32.91701	23	104.14025	32.92956
7	104.11919	32.91792	24	104.10249	32.97890
8	104.13144	32.91159	25	104.10606	32.96480
9	104.13842	32.92562	26	104.11081	32.96292
10	104.14101	32.92394	27	104.17471	32.91580
11	104.15838	32.93334	28	104.12459	32.91697
12	104.16168	32.93292	29	104.16206	32.91542
13	104.16502	32.91818	30	104.12923	32.94150
14	104.16898	32.92125	31	104.07621	32.98972
15	104.10912	32.95931	32	104.08064	32.98835
16	104.14818	32.94906	33	104.08401	32.97136
17	104.12433	32.94156			

Table S2 Location of livestock presence data.

ID	Longitude(°)	Latitude(°)	ID	Longitude(°)	Latitude(°)
1	104.11010	32.96563	35	104.05209	32.98969
2	104.11403	32.96535	36	104.07189	32.98978
3	104.11406	32.96031	37	104.03960	33.00522
4	104.07745	32.97854	38	104.02575	33.01053
5	104.07341	32.97646	39	104.02275	33.02055
6	104.06992	32.97704	40	104.04552	32.88250
7	104.06409	32.97806	41	104.02100	33.00312
8	104.06330	32.97504	42	104.00782	32.99253
9	104.07345	32.97171	43	104.00554	32.98260
10	104.13388	32.94524	44	104.01699	32.99707
11	104.12693	32.94614	45	104.05145	32.90256
12	104.12352	32.94596	46	104.05961	32.93909
13	104.12010	32.94611	47	104.05836	32.91596
14	104.11411	32.94815	48	104.05334	32.91912
15	104.10968	32.94849	49	104.06293	32.92426
16	104.09919	32.97150	50	104.06676	32.92529
17	104.10119	32.97475	51	104.13882	32.93702
18	104.10480	32.97903	52	104.14272	32.93662
19	104.10802	32.98040	53	104.14533	32.93623
20	104.11309	32.96922	54	104.14782	32.93591
21	104.11262	32.97200	55	104.15150	32.93799
22	104.15356	32.91198	56	104.06863	32.95243
23	104.12024	32.96560	57	104.10908	32.95917
24	104.12444	32.96524	58	104.16145	32.91664

25	104.12885	32.96652	59	104.14803	32.95110
26	104.15479	32.93277	60	104.03111	32.87576
27	104.16637	32.91444	61	104.02402	32.87341
28	104.17207	32.92169	62	104.00472	32.99023
29	104.14488	32.95031	63	104.08900	32.97171
30	104.12855	32.95682	64	104.08588	32.97278
31	104.12672	32.96054	65	104.08468	32.97866
32	104.13447	32.94188	66	104.08939	32.98165
33	104.13028	32.93876	67	104.09335	32.98319
34	104.12454	32.93753			

Table S3 Location of takin presence data.

ID	Longitude(°)	Latitude(°)	ID	Longitude(°)	Latitude(°)
1	104.07352	32.95765	12	104.14527	32.90657
2	104.16220	32.91630	13	104.12363	32.91708
3	104.16617	32.91565	14	104.12080	32.91756
4	104.13093	32.91258	15	104.13012	32.91603
5	104.05216	32.99257	16	104.13877	32.92609
6	104.02524	33.00055	17	104.07191	32.99072
7	104.02412	33.02205	18	104.02941	32.99874
8	104.04713	32.90265	19	104.04803	33.00123
9	104.06735	32.92614	20	104.09368	32.96302
10	104.10778	32.95881	21	104.05882	32.97696
11	104.06155	32.97863			
